# Supplementary material for: Dynamics of activation in the voltage-sensing domain of Ciona intestinalis phosphatase Ci-VSP
Source: Nat Commun. 2024 Feb 15;15:1408. doi: 10.1038/s41467-024-45514-6 (PMC10869754; doi:10.1038/s41467-024-45514-6)
Supplement: Supplementary file 1 — Supplementary Information [file 41467_2024_45514_MOESM1_ESM.pdf]

Supplementary Information

Dynamics of activation in the voltage-sensing domain of Ciona intestinalis phosphatase Ci-VSP

Spencer C. Guo,[1,2] Rong Shen,[3] Benoît Roux,[1,3,4,*)] and Aaron R. Dinner[1,4,†)]

[1)] *Department of Chemistry, The University of Chicago, Chicago, Illinois 60637*

[2)] *James Franck Institute, The University of Chicago, Chicago, Illinois 60637*

[3)] *Department of Biochemistry and Molecular Biology, The University of Chicago, Chicago, Illinois 60637*

[4)] *Institute for Biophysical Dynamics, The University of Chicago, Chicago, Illinois 60637*

SUPPLEMENTARY METHODS

Mean first passage time

Like the committor, the mean first passage time (MFPT) from a point $\mathbf{x}$ to a state B also satisfies a boundary value equation involving the stopped transition operator (that stops trajectories when they enter B):

$$m(\mathbf{x}) = \begin{cases} \mathcal{S}_t m(\mathbf{x}) = -1 & \mathbf{x} \in B^c \\ 0 & \mathbf{x} \in B \end{cases} \tag{S1}$$

For the MFPT calculations, we use an indicator basis with modified features. We construct the basis by computing the top ten eigenvectors from the 60 input features used for the committor with IVAC and lag times between 1 and 100 ns. The resulting 10-dimensional data is clustered with k-means ($k = 200$), and the resulting clusters are treated as sets that

*)Electronic mail: roux@uchicago.edu

†)Electronic mail: dinner@uchicago.edu

define indicator basis functions. We also modify the state definition of B to

$$\left(\frac{\max\{d + 4.24\text{ Å}, 0\text{ Å}\}}{1.1\text{ Å}}\right)^2 + \left(\frac{\max\{\theta + 56.95°, 0°\}}{8°}\right)^2 < 1$$

so that states beyond the up state (including the up+ state) do not contribute to the MFPT. The salt-bridge cutoffs (Supplementary Table 1) are maintained inside the original ellipse defining B. Finally, we employ a memory-based algorithm (with four memory kernels) that empirically improves the stability and accuracy of the results[1].

Backward committor

Analogously to the forward committor, the backward committor q_- represents the probability of having last exited from A, the reactant state, rather than B[2]. The backward committor also satisfies a boundary value equation involving the stopped transition operator:

$$q_-(\mathbf{x}) = \begin{cases} \mathcal{S}_{-t} q_-(\mathbf{x}) & \mathbf{x} \in (A \cup B)^c \\ 1 & \mathbf{x} \in A \\ 0 & \mathbf{x} \in B \end{cases} \tag{S2}$$

where $\mathcal{S}_{-t}$ is the backwards-in-time transition operator stopped upon first entrance into D^c. The basis set used for calculation of q_- is the same pairwise-distance basis as used for the forward committor, though the guess function is amended to $\gamma(\mathbf{x}) = d_B^2/(d_A^2 + d_B)^2$. For a reversible system (i.e., one that satisfies detailed balance, as does Ci-VSP at 0 mV), the backward committor should satisfy $q_- = 1 - q_+$, though this does not hold exactly in practice due to statistical errors in estimating committors.

When plotting committors, we use $\mathbf{E}_\pi[\mathbf{1}_{\{\xi \in ds\}}(\mathbf{x}) \mathcal{S}_\tau q_+(\mathbf{x})]$, where $\mathbf{1}_{\{\xi \in ds\}}(\mathbf{x})$ is an indicator function on a bin in our chosen CV space, e.g., $\xi(\mathbf{x}) = (d(\mathbf{x}), \theta(\mathbf{x}))$, and π is the stationary distribution, obtained by reweighting our sampling distribution μ. The propagation via $\mathcal{S}_\tau$ alleviates some of the projection error incurred in computing q_+ using DGA.

SUPPLEMENTARY TABLES

Supplementary Table 1. Salt bridge distance cutoffs for down and up states

**Salt bridge**	**Cutoff (Å)**
Down	
R226 C_ζ–D129 C_δ	> 6.0
R226 C_ζ–D186 C_δ	< 5.0
Up	
R226 C_ζ–D129 C_δ	> 5.0
R229 C_ζ–D129 C_δ	< 11.0
R229 C_ζ–D186 C_δ	< 7.5
R232 C_ζ–D186 C_δ	< 6.0

Supplementary Table 2. Collective variables considered in this work

**Type**	**Name**	**Description**
S4 helix	translocation	translocation along principal axis
	rotation	rotation around principal axis
salt bridge	R226 C_ζ-D129 C_γ	salt-bridge distances between Arg and Asp/Glu side chains
	R226 C_γ-D186 C_δ	
	R229 C_ζ-E183 C_δ	
	R229 C_ζ-D186 C_γ	
	R232 C_ζ-E183 C_δ	
	R232 C_ζ-D186 C_γ	
hydrogen bonding	R226-D129	hydrogen bonds between salt-bridge residue side chains (sensing arginines and countercharges)
	R226-D186	
	R229-E183	
	R229-D186	
	R232-E183	
	R232-D186	
hydrophobic plug	R226-F161	distances between Arg C_ζ and center-of-mass of Phe phenyl group
lipid headgroup	R217	hydrogen bonds between arginine guanidinium and phosphate groups of POPC lipids
	R229	
	R232	
arginine hydration	R223	hydrogen bonds between arginine side chain and water
	R229	
	R229	
	R232	

Supplementary Table 3. Simulation details

**Parameter**	**Value**
Initial simulation box dimensions	78 Å × 78 Å
Total number of atoms	56,582
Total number of water molecules	10,653
Salt concentration	0.1 M NaCl
Number of ions	20 Na^+, 19 Cl^-
Lipid type	POPC
Number of lipids	86 (outer leaflet), 88 (inner leaflet)

SUPPLEMENTARY FIGURES

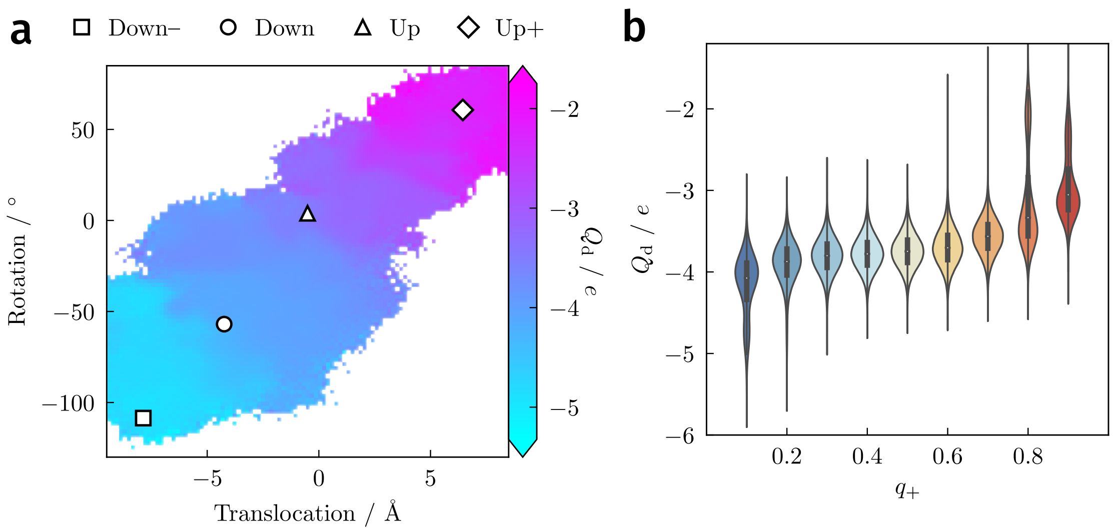

Supplementary Figure 1. Displacement charge. **a**, Average Q_d for the full system plotted against the S4 helix CVs. **b**, Distribution of displacement charge as a function of the committor. Box plots indicate median and interquartile range for each committor range. Source data are provided as a Source Data file.

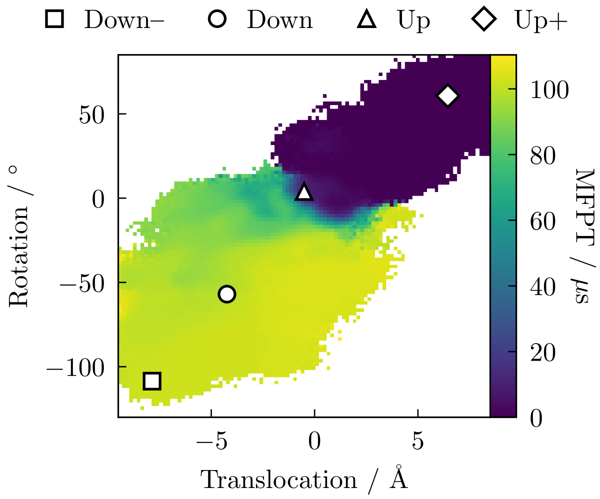

Supplementary Figure 2. Mean first-passage time (MFPT) to the up state as a function of the S4 helix CVs. The average MFPT for points in the down state is approximately 100 μs. Source data are provided as a Source Data file.

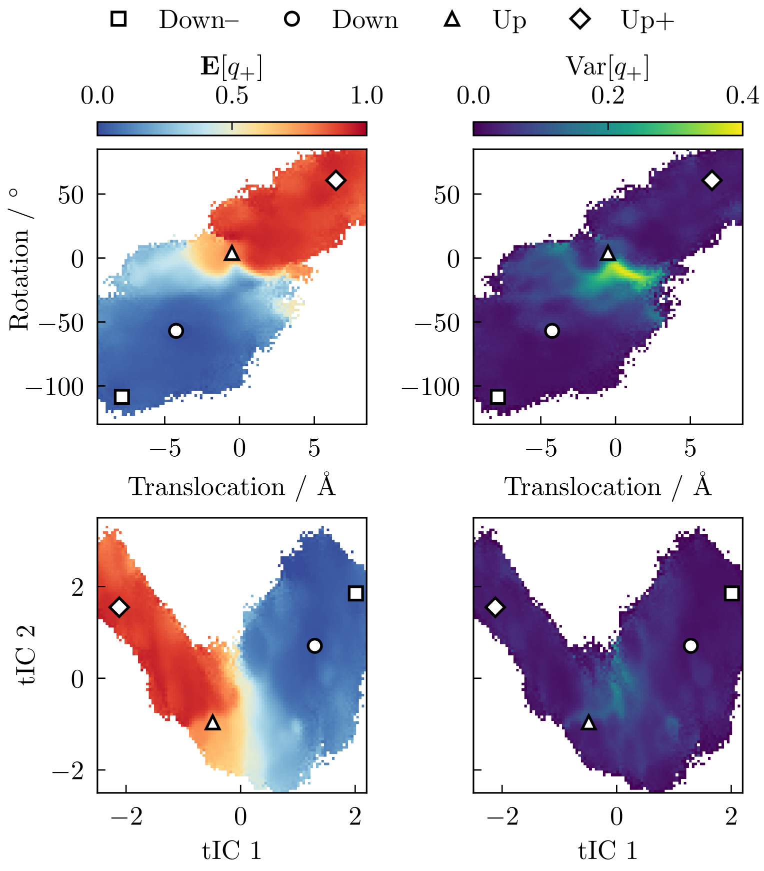

Supplementary Figure 3. Variance in committor estimates for two different CV projections. Top, S4 helix CVs. Bottom, first two IVAC coordinates. Right, the variance is computed and normalized for each CV histogram bin. Left, the average committor is reproduced from the main text for visual reference. Source data are provided as a Source Data file.

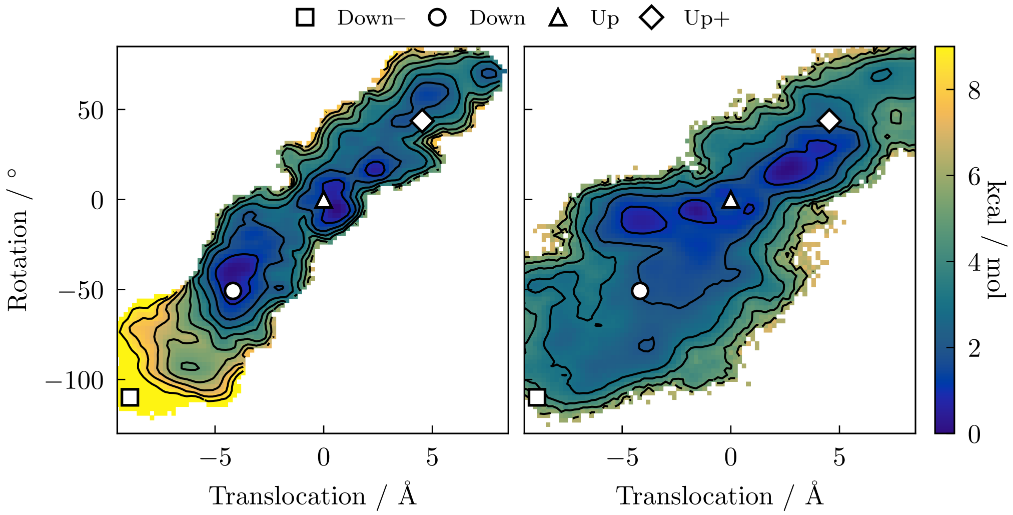

Supplementary Figure 4. Comparison of PMF along S4 helix coordinates from two different methods. (left) REUS[3] and (right) DGA. Source data are provided as a Source Data file.

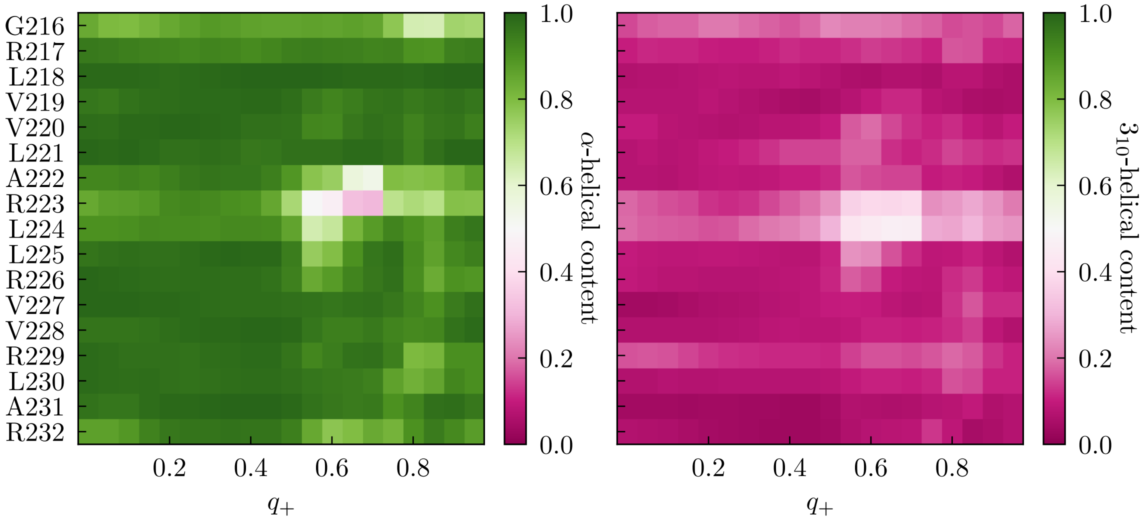

Supplementary Figure 5. Helical content of S4 residues. α-helical (left) or 3_{10}-helical (right) content of S4 residues as a function of q_+. Residue i is classified as adopting an α-helical or a 3_{10}-helical conformation if it forms a hydrogen bond ($d_{\mathrm{O-N}} < 3.5$ Å and $\angle_{\mathrm{O-H-N}} > 120°$) to residue $i+4$ or $i+3$, respectively[3].

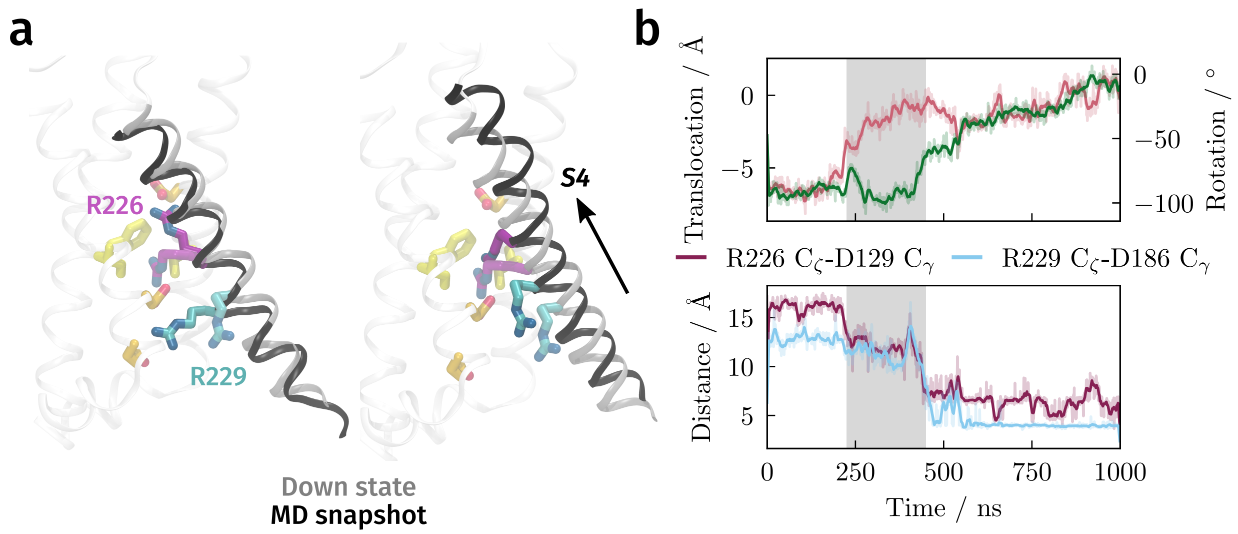

Supplementary Figure 6. Independent movements of arginine sidechains and backbone of S4 helix. **a**, Selected frames from a simulation (opaque) superimposed against the down state crystal structure (translucent) after aligning the S1–S3 helices. Only the S4 helix, R226, and R229 of the MD simulation frame are shown for clarity. **b**, Top, translocation (pink) and rotation (green) of S4 helix. Bottom, salt-bridge distances during a representative 1 μs trajectory showing translocation of the helix preceding of rotation and movement of the arginine side chains. Light lines show instantaneous values, while dark lines show moving averages over ten frames. Source data are provided as a Source Data file.

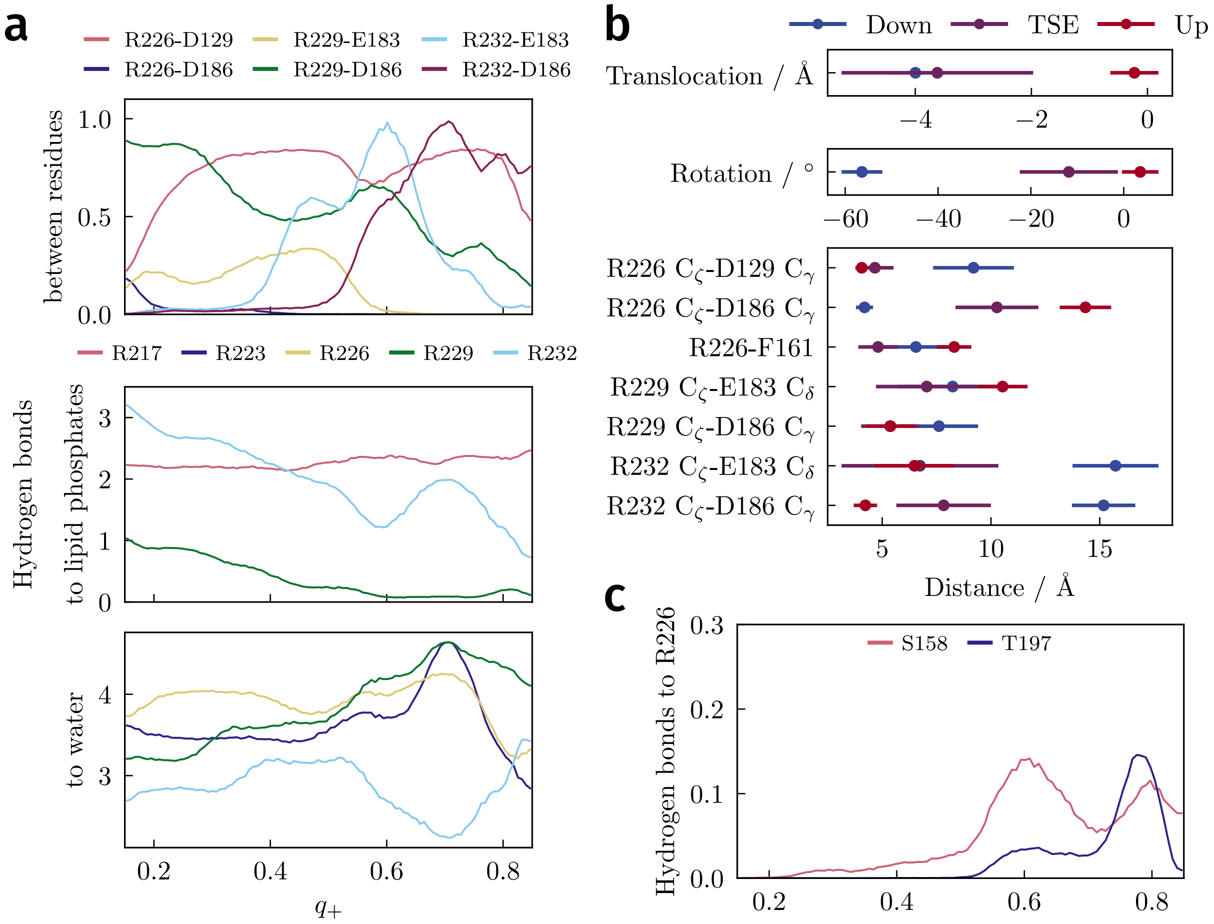

Supplementary Figure 7. Transition state ensemble and hydrogen bonding. **a**, Average number of hydrogen bonds between arginines and acidic amino acids (top), to lipid headgroups (center), and to water (bottom). Instantaneous hydrogen bond values are obtained by averaging over a moving window of 10 ns (100 frames). **b**, Average of selected CVs in the down state, in the transition state ensemble ($q_+ \in [0.45, 0.55]$), and in the up state. **c**, Average number of hydrogen bonds between R226 and S158 or T197. Source data are provided as a Source Data file.

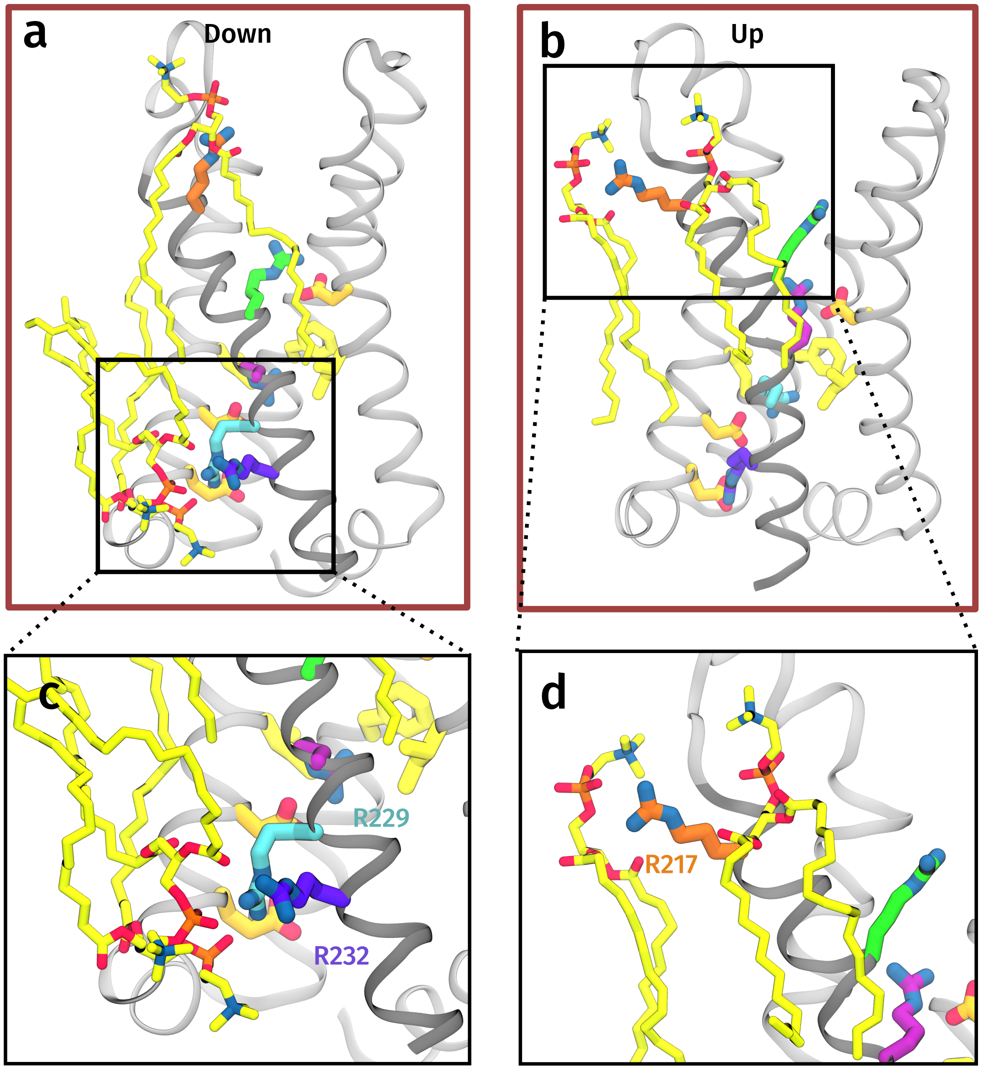

Supplementary Figure 8. Interactions between sensing arginines and lipids. **a** and **b**, Down (**a**) and up (**b**) states with nearby lipid molecules drawn. **c** and **d**, Interactions between R229/R232 in the down state (**c**) or R217 in the up state (**d**) with headgroups of POPC lipids. Phosphates and oxygens in the phosphatidylcholine headgroup are colored in orange and red, respectively, whereas carbons of lipid tails are in yellow. Source data are provided as a Source Data file.

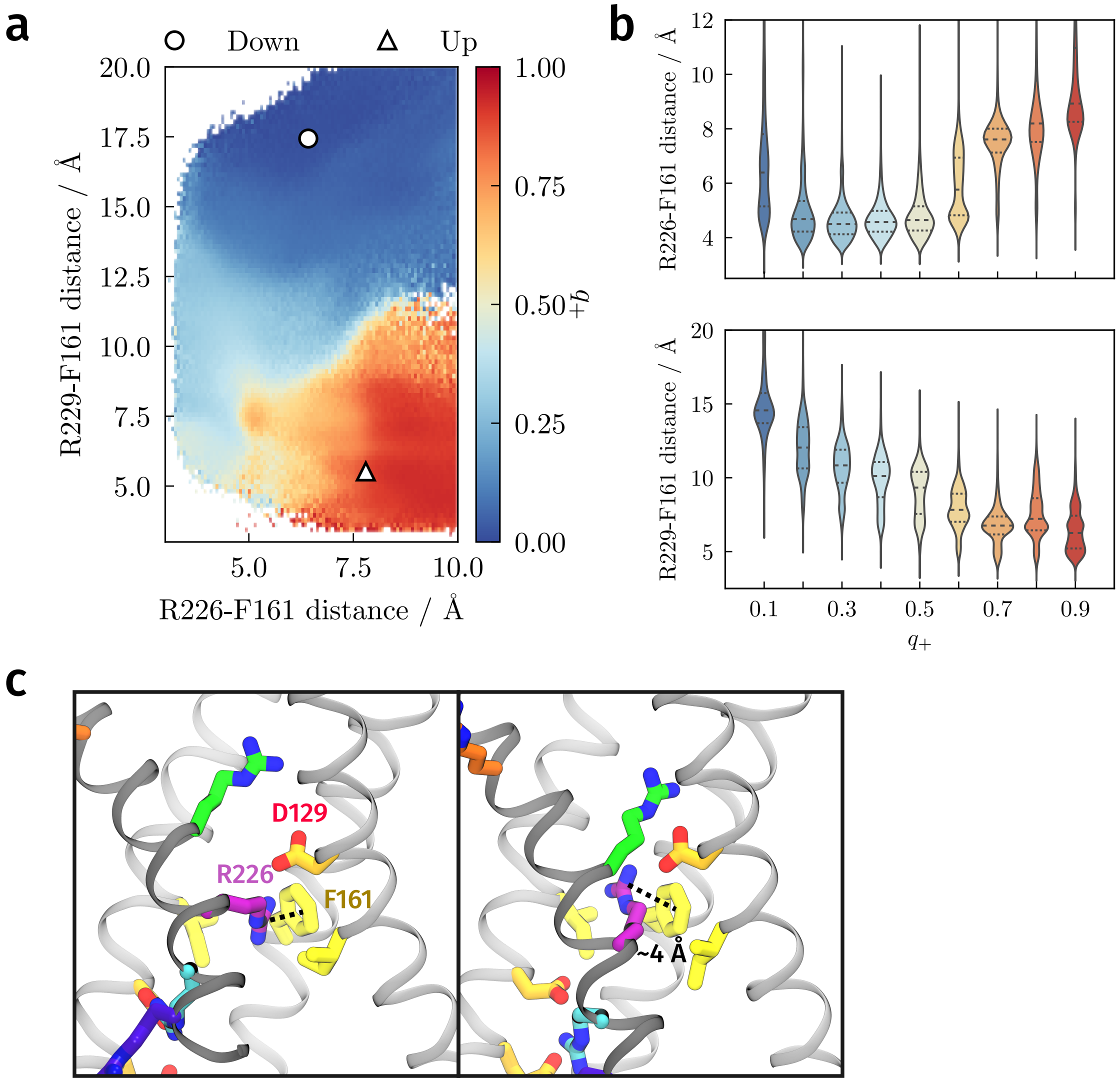

Supplementary Figure 9. Interactions between R226, R229, and F161. **a**, Committor projected onto R226-F161 and R229-F161 distances. **b**, Distribution of R226-F161 and R229-F161 distances as a function of the committor. Each violin is obtained by binning structures with the indicated $q_+ \pm 0.05$ (e.g., the first violin contains structures with $q_+ \in [0.15, 0.25]$). The median and upper/lower quartiles are denoted with dashed and solid lines, respectively. **c**, Representative configurations of R226, F161, and nearby residues. Source data are provided as a Source Data file.

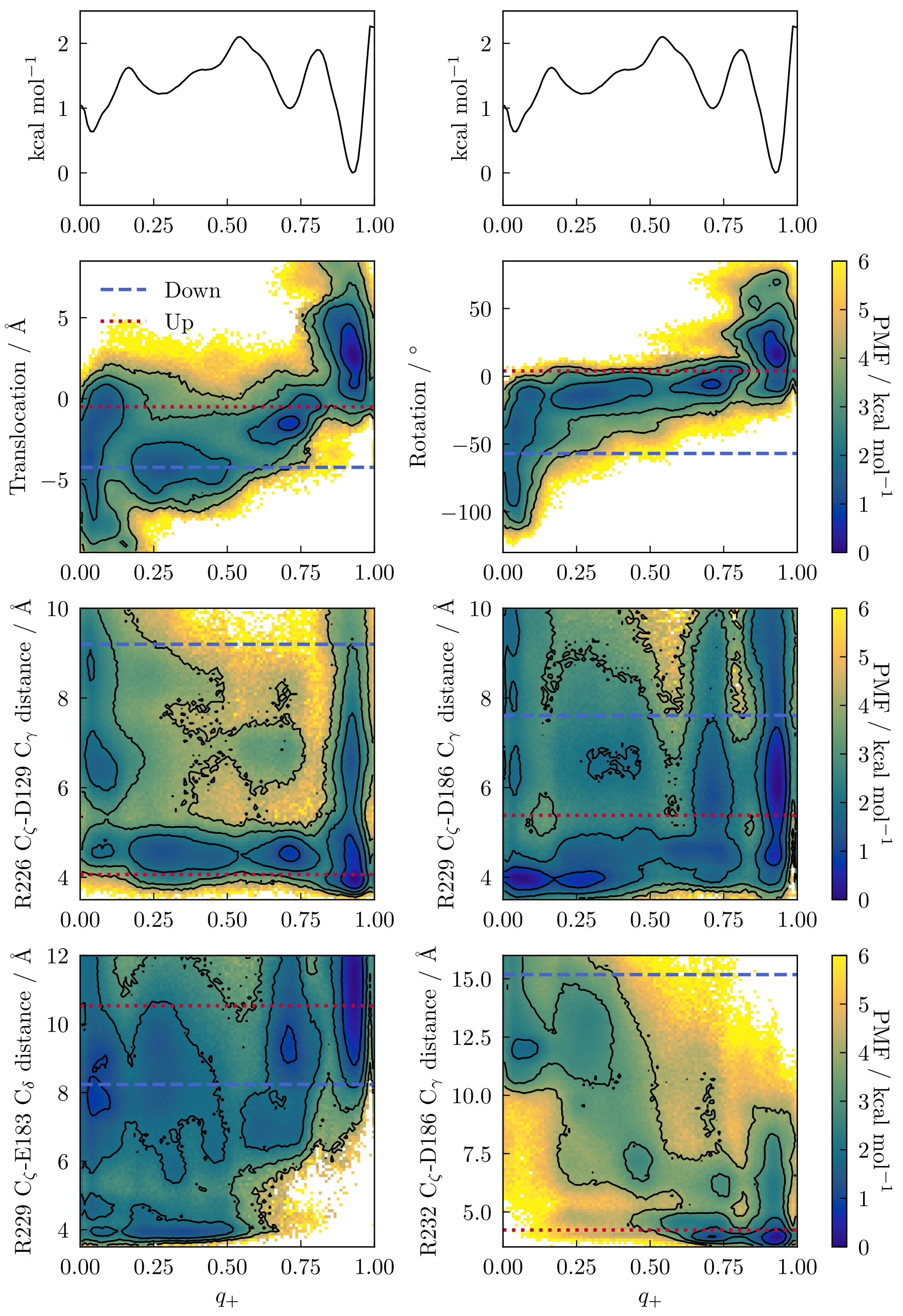

Supplementary Figure 10. PMF projected onto committor and selected CVs. CVs describe the S4 helix and salt-bridges and the projection of the PMF along the committor is shown above. Contours on the two-dimensional PMFs are drawn every 1 kcal/mol. Source data are provided as a Source Data file.

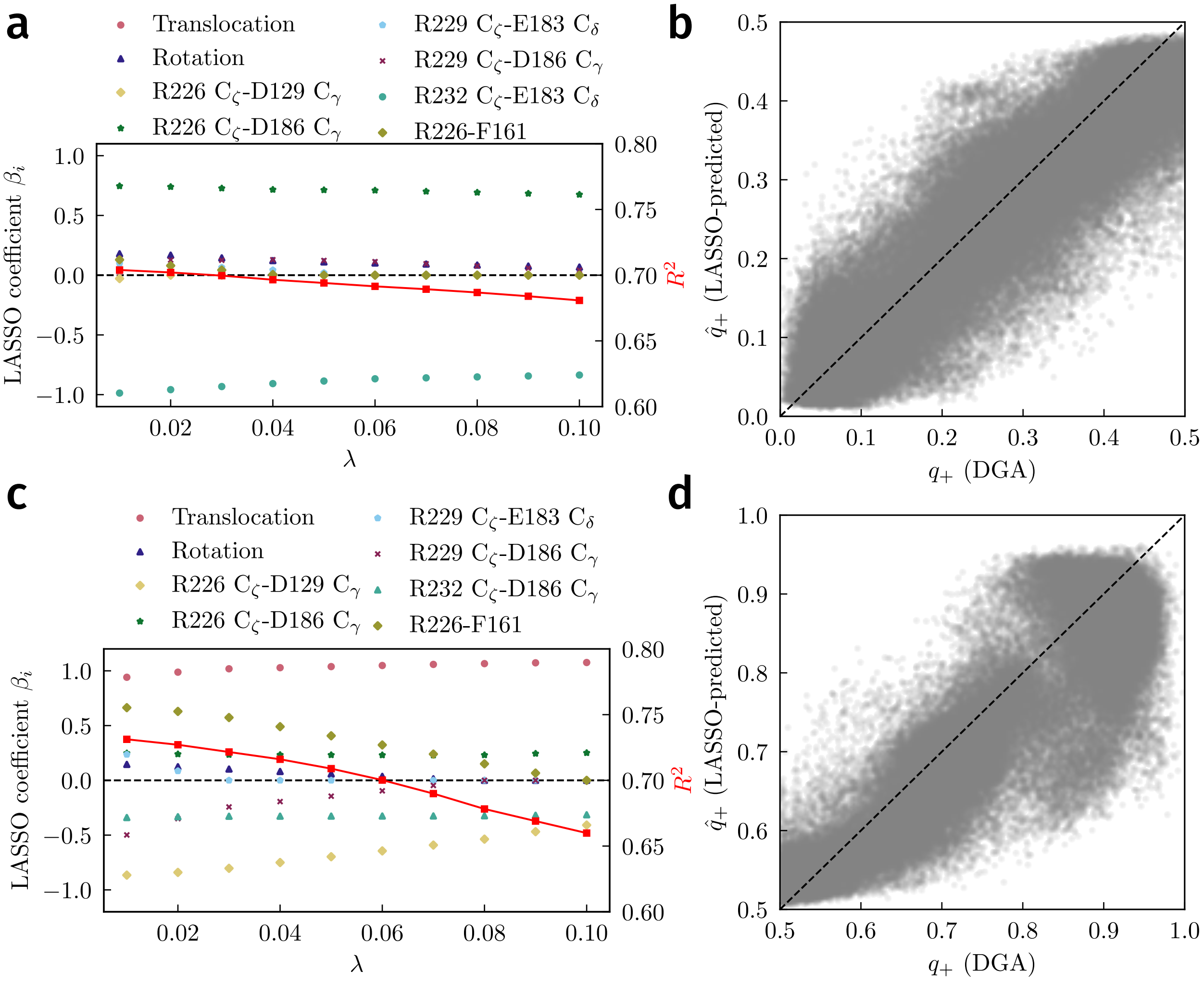

Supplementary Figure 11. Sparse regression of the committor. **a**, Changes in coefficients from LASSO fitting for the points down $\rightarrow$ TS as a function of regularization strength. Only nonzero coefficients from $\lambda = 0.02$ are shown. The goodness-of-fit, R^2 is plotted as red squares. **b**, Scatter plot of DGA committors versus LASSO committors. **c**, Changes in coefficients from LASSO fitting for the points TS $\rightarrow$ up as a function of regularization strength. Only nonzero coefficients from $\lambda = 0.03$ are shown. The goodness-of-fit, R^2 is plotted as red squares. **d**, Scatter plot of DGA committors versus LASSO committors. Source data are provided as a Source Data file.

SUPPLEMENTARY REFERENCES

[1]Lorpaiboon, C., Guo, S. C., Strahan, J., Weare, J. & Dinner, A. R. Accurate estimates of dynamical statistics using memory (2023). ArXiv:2311.07881 [cond-mat, physics:physics].

[2]Vanden-Eijnden, E. Transition path theory. In *Computer Simulations in Condensed Matter Systems: From Materials to Chemical Biology Volume 1*, 453–493 (Springer, 2006).

[3]Shen, R., Meng, Y., Roux, B. & Perozo, E. Mechanism of voltage gating in the voltage-sensing phosphatase Ci-VSP. *Proc. Natl Acad. Sci. USA* **119**, e2206649119 (2022).
