# Supplementary material for: Dynamics of activation in the voltage-sensing domain of Ciona intestinalis phosphatase Ci-VSP
Source: Nat Commun. 2024 Feb 15;15:1408. doi: 10.1038/s41467-024-45514-6 (PMC10869754; doi:10.1038/s41467-024-45514-6)
Supplement: Supplementary file 3 — Description of Additional Supplementary Files [file 41467_2024_45514_MOESM3_ESM.pdf]

File name: Supplementary Movie 1

Description: Down-up transition in Ci-VSD. Five configurations are sampled from twenty equally spaced windows of the committor (i.e., of width 0.05) and are aligned. The red line indicates the progress of the transition along the committor.
