# Supplementary material for: Dynamics of activation in the voltage-sensing domain of Ciona intestinalis phosphatase Ci-VSP
Source: Nat Commun. 2024 Feb 15;15:1408. doi: 10.1038/s41467-024-45514-6 (PMC10869754; doi:10.1038/s41467-024-45514-6)
Supplement: Supplementary file 5 — Reporting Summary [file 41467_2024_45514_MOESM5_ESM.pdf]

Corresponding author(s): Aaron Dinner, Benoit Roux

Last updated by author(s): Dec 8, 2023

Reporting Summary

Nature Portfolio wishes to improve the reproducibility of the work that we publish. This form provides structure for consistency and transparency in reporting. For further information on Nature Portfolio policies, see our Editorial Policies and the Editorial Policy Checklist.

Statistics

For all statistical analyses, confirm that the following items are present in the figure legend, table legend, main text, or Methods section.

n/a	Confirmed	
☐	☒	The exact sample size (*n*) for each experimental group/condition, given as a discrete number and unit of measurement
☒	☐	A statement on whether measurements were taken from distinct samples or whether the same sample was measured repeatedly
☒	☐	The statistical test(s) used AND whether they are one- or two-sided<br>*Only common tests should be described solely by name; describe more complex techniques in the Methods section.*
☒	☐	A description of all covariates tested
☒	☐	A description of any assumptions or corrections, such as tests of normality and adjustment for multiple comparisons
☐	☒	A full description of the statistical parameters including central tendency (e.g. means) or other basic estimates (e.g. regression coefficient) AND variation (e.g. standard deviation) or associated estimates of uncertainty (e.g. confidence intervals)
☒	☐	For null hypothesis testing, the test statistic (e.g. *F*, *t*, *r*) with confidence intervals, effect sizes, degrees of freedom and *P* value noted<br>*Give P values as exact values whenever suitable.*
☒	☐	For Bayesian analysis, information on the choice of priors and Markov chain Monte Carlo settings
☒	☐	For hierarchical and complex designs, identification of the appropriate level for tests and full reporting of outcomes
☒	☐	Estimates of effect sizes (e.g. Cohen's *d*, Pearson's *r*), indicating how they were calculated

Our web collection on statistics for biologists contains articles on many of the points above.

Software and code

Policy information about availability of computer code

Data collection	Molecular dynamics simulation data were produced using Anton 2 (code not publicly available; resource handled through Pittsburgh Supercomputing Center, https://www.psc.edu/resources/anton-2/) and AMBER20 with PLUMED 2.7.
Data analysis	Analysis of molecular dynamics data was performed with PyEMMA 2.5.12, MDTraj 1.9.7, MDAnalysis 2.4.2, scikit-learn 1.2.1, and VMD 1.9.4 along with custom codes written in Python/Jupyter notebooks and tcl. Custom codes are available at https://github.com/dinner-group/ci-vsd.

For manuscripts utilizing custom algorithms or software that are central to the research but not yet described in published literature, software must be made available to editors and reviewers. We strongly encourage code deposition in a community repository (e.g. GitHub). See the Nature Portfolio guidelines for submitting code & software for further information.

Data

Policy information about availability of data

All manuscripts must include a data availability statement. This statement should provide the following information, where applicable:

- Accession codes, unique identifiers, or web links for publicly available datasets
- A description of any restrictions on data availability
- For clinical datasets or third party data, please ensure that the statement adheres to our policy

The PDB files for the down and up states of Ci-VSD are available at wwPDB with accession codes 4G80 and 4G7V, respectively. The trajectory data without lipid or water coordinates, together with simulation parameters and initial and final structures of trajectories have been deposited in Zenodo under accession code https://

doi.org/10.5281/zenodo.10231877. The raw simulation data have not been deposited due to their size; access can be obtained by contacting the authors or the Pittsburgh Supercomputing Center (PSC).The processed data (CVs, DGA quantities) generated in this study are provided in the Source Data file.

Research involving human participants, their data, or biological material

Policy information about studies with human participants or human data. See also policy information about sex, gender (identity/presentation), and sexual orientation and race, ethnicity and racism.

Reporting on sex and gender	N/A
Reporting on race, ethnicity, or other socially relevant groupings	N/A
Population characteristics	N/A
Recruitment	N/A
Ethics oversight	N/A

Note that full information on the approval of the study protocol must also be provided in the manuscript.

Field-specific reporting

Please select the one below that is the best fit for your research. If you are not sure, read the appropriate sections before making your selection.

☒ Life sciences ☐ Behavioural & social sciences ☐ Ecological, evolutionary & environmental sciences

For a reference copy of the document with all sections, see nature.com/documents/nr-reporting-summary-flat.pdf

Life sciences study design

All studies must disclose on these points even when the disclosure is negative.

Sample size	No method was use to predetermine the sample size. Convergence of simulations was assessed via visualization of the quantities computed (e.g. committors) as the number of trajectories was increased.
Data exclusions	No data were excluded from the study.
Replication	No specific steps were undertaken to replicate the study. However, a large sample size of initial conditions (352) for simulations was used to reduce the effects of stochasticity. The qualitative findings remained consistent when a fraction of the simulation data were withheld.
Randomization	Randomization was not applicable to this study as the goal was not to compare the effect of different experimental conditions.
Blinding	Blinding was not applicable to this study as simulation data are not affected by the analysis.

Reporting for specific materials, systems and methods

We require information from authors about some types of materials, experimental systems and methods used in many studies. Here, indicate whether each material, system or method listed is relevant to your study. If you are not sure if a list item applies to your research, read the appropriate section before selecting a response.

Materials & experimental systems

n/a	Involved in the study
☒	☐ Antibodies
☒	☐ Eukaryotic cell lines
☒	☐ Palaeontology and archaeology
☒	☐ Animals and other organisms
☒	☐ Clinical data
☒	☐ Dual use research of concern
☒	☐ Plants

Methods

n/a	Involved in the study
☒	☐ ChIP-seq
☒	☐ Flow cytometry
☒	☐ MRI-based neuroimaging

Plants

Seed stocks	N/A
Novel plant genotypes	N/A
Authentication	N/A
